# Which cardiac parameters best predict the cardiovascular outcomes among patients with anti-PD-1 immunotherapy-induced myocardial injury?

**DOI:** 10.3389/fcvm.2022.922095

**Published:** 2022-09-23

**Authors:** Xiongjun Peng, Yawen Zheng, Zhaowei Zhu, Na Liu, Shenghua Zhou, Junke Long

**Affiliations:** ^1^The Second Xiangya Hospital, Central South University, Changsha, China; ^2^National Clinical Research Center for Geriatric Disorders, Department of Geriatrics, Xiangya Hospital, Central South University, Changsha, China; ^3^Department of Cardiovascular Medicine, The Second Xiangya Hospital, Central South University, Changsha, China

**Keywords:** cardiac parameters, anti-PD-1 immunotherapy, myocardial injury, prognostic predictor, cardiogenic death

## Abstract

**Aim:**

To explore the association of cardiac parameters with different clinical outcomes in patients with anti-PD-1 immunotherapy-induced myocardial injury.

**Methods and results:**

We screened 3,848 patients who received anti-PD-1 immunotherapy from June 2018 to Oct 2021 at the Second Xiangya Hospital of Central South University. Among those patients, 134 patients were diagnosed with anti-PD-1 immunotherapy-induced myocardial injury. Twenty-four patients with cardiovascular symptoms were divided into the major adverse cardiac events (MACE) group, and 110 patients without cardiovascular symptoms were divided into the non-MACE group. We compared creatine kinase isozyme (CK-MB), high-sensitivity troponin T (hsTNT), N-terminal pro–B-type natriuretic peptide (NT-ProBNP), electrocardiography (ECG), and echocardiographic parameters between the two groups of patients. CK-MB, hsTNT, NT-proBNP [2,600.0 (1,317.00–7,950.00) vs. 472.9 (280.40–788.80), *p* ≤ 0.001], left ventricular end-diastolic diameter (LVEDd), left ventricular ejection fraction (LVEF) and QRS interval were significantly different. The receiver operating characteristic (ROC) curve was used to compare the accuracy of various indicators to predict the occurrence of MACE events. NT-ProBNP (area under the curve [*AUC*] 97.1) was the best predictor, followed by CK-MB (*AUC* = 94.1), LVEF (*AUC* = 83.4), LVEDd (*AUC* = 81.5), and other indicators. In the MACE group, 11/24 patients had experienced cardiogenic death by the end of follow-up. There were significant differences in the CK-MB, hsTNT, NT-proBNP, LVEDd, LVEF, and QRS intervals between the deceased patients and the survivors. The ROC curve shows that hsTNT is the most accurate marker for predicting cardiogenic death in the MACE group (*AUC* = 91.6).

**Conclusion:**

In patients with myocardial injury after PD-1 inhibitor treatment, NT-proBNP is the parameter of choice to predict the likelihood of developing cardiovascular symptoms, whereas, in symptomatic patients, hsTNT is the optimal parameter associated with the outcome of death compared with other cardiac parameters.

## Introduction

Cancer and cardiovascular diseases are the two most important categories of diseases affecting human health (1). Immunotherapy has advanced rapidly in the treatment of tumors in recent decades (2). In particular, immune checkpoint inhibitors (ICIs) represented by anti-programmed cell death-1 (PD-1) antibody therapy are one of the most commonly used immunotherapy methods worldwide (3). According to the guidelines published by multiple oncology organizations around the world (4, 5), PD-1 inhibitors have become a standard treatment for a variety of solid advanced malignancies, and immunotherapy-induced myocardial injury has increasingly been recognized with the widespread use of these agents (6). Some patients have only isolated elevation of serum markers of myocardial injury without any complaints, such as creatine kinase isozyme (CK-MB), high-sensitivity troponin T (hsTnT), and N-terminal pro–B-type natriuretic peptide (NT-proBNP). However, some patients treated with anti-PD-1 inhibitors also have severe cardiovascular manifestations, such as heart failure (HF), malignant arrhythmias, and death, even though a lower incidence of 0.3–2% has been reported in the literature (7, 8). The mechanism leading to this completely different clinical outcome is not yet been fully understood and may be related to the excessive activation of inflammation. In addition, whether patients with asymptomatic myocardial injury need treatment is unclear. Regardless, it is foreseeable that the use of ICIs will continue to increase as the cost decreases, and therefore, how to accurately identify the severity of PD-1 inhibitor-induced myocardial injury at an early stage is of great importance but remains unclear.

Our objective was to identify the association of cardiac parameters with different clinical outcomes in patients with anti-PD-1 immunotherapy-induced myocardial injury and find a better cardiac parameter to predict these outcomes of different severities.

## Patients and methods

### Patients

This is a retrospective cohort study, we screened 3,848 patients who received anti-PD-1 immunotherapy from June 2018 to Oct 2021 at the Second Xiangya Hospital of Central South University. Among those patients, 134 patients were diagnosed with anti-PD-1 immunotherapy-induced myocardial injury. These patients were from the Department of Oncology, Department of Respiratory Medicine, Department of Cardiology, Department of Thoracic Surgery, Department of Critical Medicine, and Department of Emergency. Medical records are from the inpatient, outpatient, and emergency medical systems. Data including demographic characteristics, comorbidities, main complaint at diagnosis, laboratory testing results, electrocardiography (ECG), echocardiographic findings, and treatment were obtained. The study protocol conformed to the ethical guidelines of the Declaration of Helsinki (9) as reflected by prior approval from the human research committee of the Second Xiangya Hospital of Central South University. Written informed consent was obtained from patients while the patient was in a clinically stable, non-congested condition or from their family members who could give informed consent on behalf of patients after they were informed about the objectives and procedures of the study. Their rights to refuse participation any time they wanted were assured. For this purpose, a one-page consent letter was attached as a cover page of each questionnaire stating the general objective of the study and issues of confidentiality that were discussed by the data collectors before proceeding with the data collection.

### Diagnostic procedures

**Figure 1:**
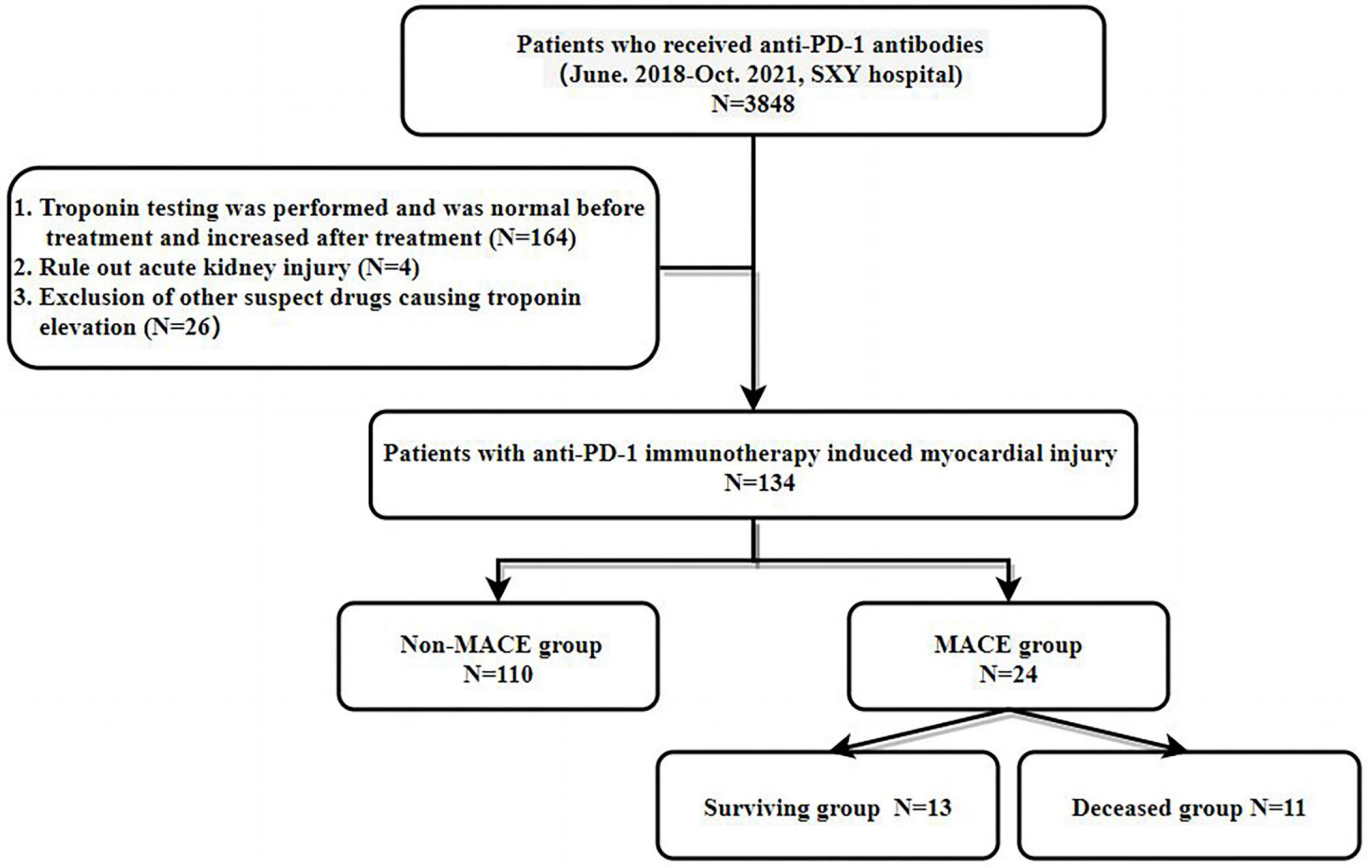
Flowchart of the present study.

A total of 3,848 patients who received PD-1 antibody therapy were evaluated. The inclusion criteria were as follows: (1) High-sensitivity troponin T was negative in patients before PD-1 antibody treatment, but the concentration increased in patients after treatment. The exclusion criteria were: (1) Acute or suspected renal function injury leading to false elevation of high-sensitivity troponin T levels; and (2) Use of other drugs that may cause myocardial injuries, such as anthracycline chemotherapeutic drugs, cyclophosphamide, and trastuzumab. Finally, 134 patients were included in the study (see flowchart in Figure 1 for details).

### Data collection

Demographic and clinical characteristics were collected when the diagnosis of PD-1 inhibitor-induced myocardial injury was confirmed. Blood test parameters, ECG, and cardiac ultrasound parameters were the first reports obtained after diagnosis. Patient-reported comorbidities were listed according to what the patient told our doctor on admission and what we diagnosed after discharge. The tracking of hsTNT and NT-proBNP is generally divided into two situations. The first is that it is detected in our hospital when symptoms appear. The second is the routine detection of asymptomatic patients on admission for anti-tumor treatment. High-sensitivity troponin T (hsTnT) was measured by electrochemiluminescence (Roche, Germany). The upper limit of the reference value (99th quantile) in the manual is 14 pg/ml.

### Definitions and outcome

The outcome of interest, major adverse cardiac events (MACE), was a composite of cardiovascular death, cardiac arrest, HF, and arrhythmias that cause hemodynamic abnormalities such as tachyarrhythmias/bradyarrhythmias and acute myocardial infarction (AMI). For cases where cardiac arrest, HF, arrhythmias, and AMI led to death, the outcome was counted as cardiac death. Standard definitions were used for cardiovascular death, cardiac arrest, HF, and AMI (10, 11). Survival time (days) was measured as the duration between the first day of hospitalization when the patient received PD-1 antibody therapy to the date of MACE or death from any cause. Data were obtained from medical records or from telephone interviews with patients or relatives by 2 trained physicians. We chose to set the follow-up time to 90 days because previous clinical studies showed that the vast majority of cardiotoxicity occurred within 90 days following the use of PD-1 inhibitors (12). Patients were followed until 16 October 2021. Patients were censored if they were still alive at the end of the research period or were lost to follow-up, on which occasion their last clinic visit or correspondence time was used.

### Statistical analysis

Normally distributed parameters are expressed as the mean ± standard deviation (*SD*), whereas non-normally distributed parameters are expressed as the median with interquartile range (*IQR*). Categorical values are presented as numbers (percentages). Categorical data were reported as frequencies and percentages and were compared using the chi-squared or Fisher's exact test. Comparison of continuous variables between two independent groups was performed using an unpaired Student's *t*-test (if normally distributed) or the Mann–Whitney *U* test (non-normally distributed variables), and in cases where more than two groups were compared, one-way analysis of variance (ANOVA) or the Kruskal–Wallis test was used. Univariate analyses were performed to examine the correlates between cardiac parameters and different outcomes using the logistic regression models. The receiver operating characteristic (ROC) curve was used to reflect the accuracy of different cardiac parameters in predicting different outcomes by the area under the curve (AUC). Survival was evaluated with Kaplan–Meier curves. All tests were two-tailed and a *p*-value of < 0.05 was considered to indicate statistically significant. Statistical analysis was performed using SPSS 26.0 (IBM Software Inc), EmpowerStats 3.0 software, and R (version 3.3.2).

## Results

### Patient characteristics

In the MACE group, 16 patients had a new onset HF, 3 patients had non-ST segment elevation myocardial infarction (NSTEMI), 3 patients had new-onset symptomatic arrhythmia, and 1 patient had a sudden cardiac arrest. In the non-MACE group (*n* = 110), no patients presented with clinical symptoms of the cardiovascular system. Although high-sensitivity troponin T or NT-proBNP levels were significantly higher than before PD-1 antibody administration. The MACE group was older than the non-MACE group (66.5 ± 8.1 vs. 60.4 ± 9.9 *p* = 0.01). In addition, the MACE group had more concurrent side effects, such as PD-1-mediated pneumonia [7/24 (29.2%) vs. 1/110 (0.9%)], hepatitis [3/24 (12.5%) vs. 3/110 (2.7%)], myositis [4/24 (16.7%) vs. 3/110 (2.7%)], and thyroid dysfunction [5/24 (20.8%) vs. 16/110 (14.5%)]. Regarding sex, 75% (18/24) of patients in the MACE group were male, and 78.2% (86/110) in the non-MACE group were male. There was no significant difference in the gender distribution between the two groups (Table 1).

**Table 1:** Characteristics of 134 patients with programmed cell death (PD-1)-related myocardial injury.

	**No MACE (*n* = 110)**	**MACE (*n* = 24)**	***P*-value**
Age, years	60.4 (9.9)	66.5 (8.1)	0.010
Male, *n* (%)	86(78.2)	18 (75)	0.735
SBP, mmHg	115.48 (21.13)	119.25 (17.52)	0.233
DBP, mmHg	70.45 (11.57)	70.46 (13.47)	0.738
**NYHA**, ***n*** **(%)**			< 0.001
Class I–II	110(100)	9(37.5)	
Class III–IV	–	15(62.5)	
SpO_2_, %	96.45 (2.43)	96.25 (2.72)	0.962
TPS, %	47.35 (27.51)	44.12 (27.23)	0.654
Days from first dose	32.85 (17.97)	37.04 (20.26)	0.257
**Primary cancer type**, ***n*** **(%)**			0.822
Lung cancer	66 (60)	16 (66.7)	
Esophageal cancer	14 (12.7)	2 (8.3)	
Liver cancer	10 (9.1)	3 (12.5)	
Other tumors	20 (18.2)	3 (12.5)	
**Comorbidities**, ***n*** **(%)**			0.356
COPD	15 (13.6)	5 (20.8)	
Hypertension	38 (34.5)	8 (33.3)	
Hyperlipidemia	28 (25.5)	6 (25)	
CKD	22 (20)	5 (20.8)	
T2DM	16 (14.6)	2 (8.4)	
Stroke	15 (13.6)	2 (8.3)	
CHD	16 (14.5)	6 (25)	
**Anti-tumor regimen**, ***n*** **(%)**			0.188
PD-1 monotherapy	40(36.4)	7 (29.2)	
Combined chemotherapy	70 (63.6)	17 (70.8)	
**Concurrent side effects**, ***n*** **(%)**			0.001
Pneumonitis	1 (0.9)	7 (29.2)	
Hepatitis	3 (2.7)	3 (12.5)	
Thyroid dysfunction	16 (14.5)	5 (20.8)	
Myositis	3 (2.7)	4 (16.7)	
**Baseline cardiac parameters**			
Cardiac troponin T, pg/mL	8.0 (6.3–10.2)	7.6 (5.3–9.8)	0.285
PR interval, ms	154.9 ± 17.3	161.2 ± 36.4	0.214
Corrected QT interval, ms	452.6 ± 36.2	448.5 ± 52.2	0.654
QRS duration, ms	95.2 ± 19.4	87.0 ± 17.1	0.058
**Baseline cardiovascular medications**			
Aspirin	15 (13.6%)	5 (20.8%)	0.370
ACEI or ARB	10 (9.1%)	4 (16.7%)	0.272
βblockers	11 (10.0%)	4 (16.7%)	0.348

Data are (N) Mean (SD) or (N) n (%), Median (Q3–Q1), where N is the total number of patients with available data. SBP, Systolic Blood Pressure; DBP, Diastolic Blood Pressure; SpO_2_, Saturation of Peripheral Oxygen; TPS, Tumor Proportion Score; NYHA, New York Heart Association Functional Classification; CHD, Coronary Heart Disease; COPD, Chronic Obstructive Pulmonary Disease; CKD, Chronic Kidney Disease; T2DM, Type 2 Diabetes Mellitus; PD-1,Programmed Cell Death.

### Cancer characteristics of interest

The time from the first day of PD-1 inhibitor treatment to the date when PD-1 inhibitor-induced myocardial injury diagnosis was confirmed was 37.04 ± 20.26 days for the MACE group and 32.85 ± 17.97 days for the non-MACE group. Regarding the tumor proportion scores (TPS) of PD-1 expression by tumor tissue immunohistochemistry, there was no difference between the two groups (47.35 ± 27.51 vs. 44.12 ± 27.23, *p* = 0.654). Regarding the anti-tumor regimen, 7 (29.2%) patients in the MACE group and 40 (36.4%) patients in the non-MACE group received PD-1 inhibitor monotherapy. The remaining cases were treated with chemotherapy combined with immunotherapy. More than half of the patients' primary tumors were non-small-cell lung cancer (NSCLC), followed by esophageal cancer, liver cancer, and other tumors (details in Table 1).

### Cardiac parameters among subjects

In the MACE group, CK-MB (108.97 ± 57.09 vs. 31.86 ± 43.66, *p* ≤ 0.001), hsTNT [195.5 (108.75–302.50) vs. 78.00 (47.85–124.00), *p* ≤ 0.001], and NT-proBNP [2,600.0 (1,317.00–7,950.00) vs. 472.9 (280.40–788.80), *p* ≤ 0.001] levels were significantly higher than those in the non-MACE group. Regarding the parameters of echocardiography, in the MACE group, patients had a higher left ventricular end-diastolic diameter (LVEDd) (51.5 ± 6.1 vs. 43.5 ± 6.2, *p* ≤ 0.001) and lower left ventricular ejection fraction (LVEF) (46.7 ± 9.1 vs. 57.2 ± 7.5, *p* ≤ 0.001) than those in the non-MACE group. There were no significant differences in other cardiac parameters between the two groups (Table 2). The ECG parameters between the two groups were also somewhat different. The incidence of bradyarrhythmia and tachyarrhythmia in the MACE group was higher than that in the non-MACE. The QRS interval of the MACE group was significantly wider than that of the non-MACE group (127.2 ± 33.5 vs. 93.7 ± 16.1, *p* = 0.001), but the corrected QT interval of the two groups was no different (details in Table 2).

**Table 2:** Laboratory, echocardiographic, and electrocardiographic characteristics and treatment of 134 patients with PD-1-related myocardial injury.

	**No MACE (*n* = 110)**	**MACE (*n* = 24)**	***P*-value**
**Laboratory results**			
CK-MB, u/L	31.86 (43.66)	108.97 (57.09)	**< 0.001**
Cardiac troponin T, pg/mL	78.00 (47.85–124.00)	195.5 (108.75–302.50)	**< 0.001**
NT-proBNP, pg/mL	472.9 (280.40–788.80)	2600.0(1317.00–7950.00)	**< 0.001**
**Echocardiographic findings**			
LVEDd, mm	43.5 (6.2)	51.5 (6.1)	**< 0.001**
RVEDd, mm	33.7 (4.3)	33.0(4.3)	0.380
LAESd, mm	36.5 (5.9)	37.4 (5.0)	0.201
RAESd, mm	34.1 (5.3)	33.5 (5.1)	0.614
LVEF, (%)	57.2 (7.5)	46.7 (9.1)	**< 0.001**
**ECG findings**			
Atrial fibrillation, *n* (%)	11 (10)	1 (4.2)	0.693
Advanced AV block, *n* (%)	2 (1.8)	8 (33.3)	**0.001**
Bundle branch block	24 (21.8)	7 (29.2)	0.256
FVP or VT, *n* (%)	19 (17.3)	11 (45.8)	**0.006**
PR interval, ms	170.6 (32.2)	168.3 (26.2)	0.929
Corrected QT interval, ms	457.3 (34.1)	470.5 (35.4)	0.173
QRS duration, ms	93.7 (16.1)	127.2 (33.5)	**< 0.001**
**Therapeutic cardiovascular medications**			
Aspirin	16 (14.5)	8 (33.3)	**0.040**
ACEI or ARB	12 (10.9)	10 (41.7)	0.010
βblockers	16 (14.5)	5 (20.8)	0.743
Furosemide	2 (1.8)	17 (70.8)	**< 0.001**
Inotropic agents	0 (0)	6 (25)	**< 0.001**
Glucocorticoid	1 (0.9)	13 (54.2)	**< 0.001**

Data are (N) Mean (SD) or (N) n (%), Median (Q3–Q1), where N is the total number of patients with available data. CK-MB, Creatine Kinase isoenzyme MB; NT-proBNP, N-terminal pro–B-type Natriuretic Peptide; LAESd, Left Atrium End Systolic diameter; LVEDd, Left Ventricular End Diastolic diameter; RAESd, Right Atrium End Systolic diameter; RVEDd, Right Ventricular End Diastolic diameter; LVEF, Left Ventricular Ejection Fraction; FVP, Frequent Ventricular Premature; VT, Ventricular Tachycardia.

### Outcome of all cases

**Figure 2:**
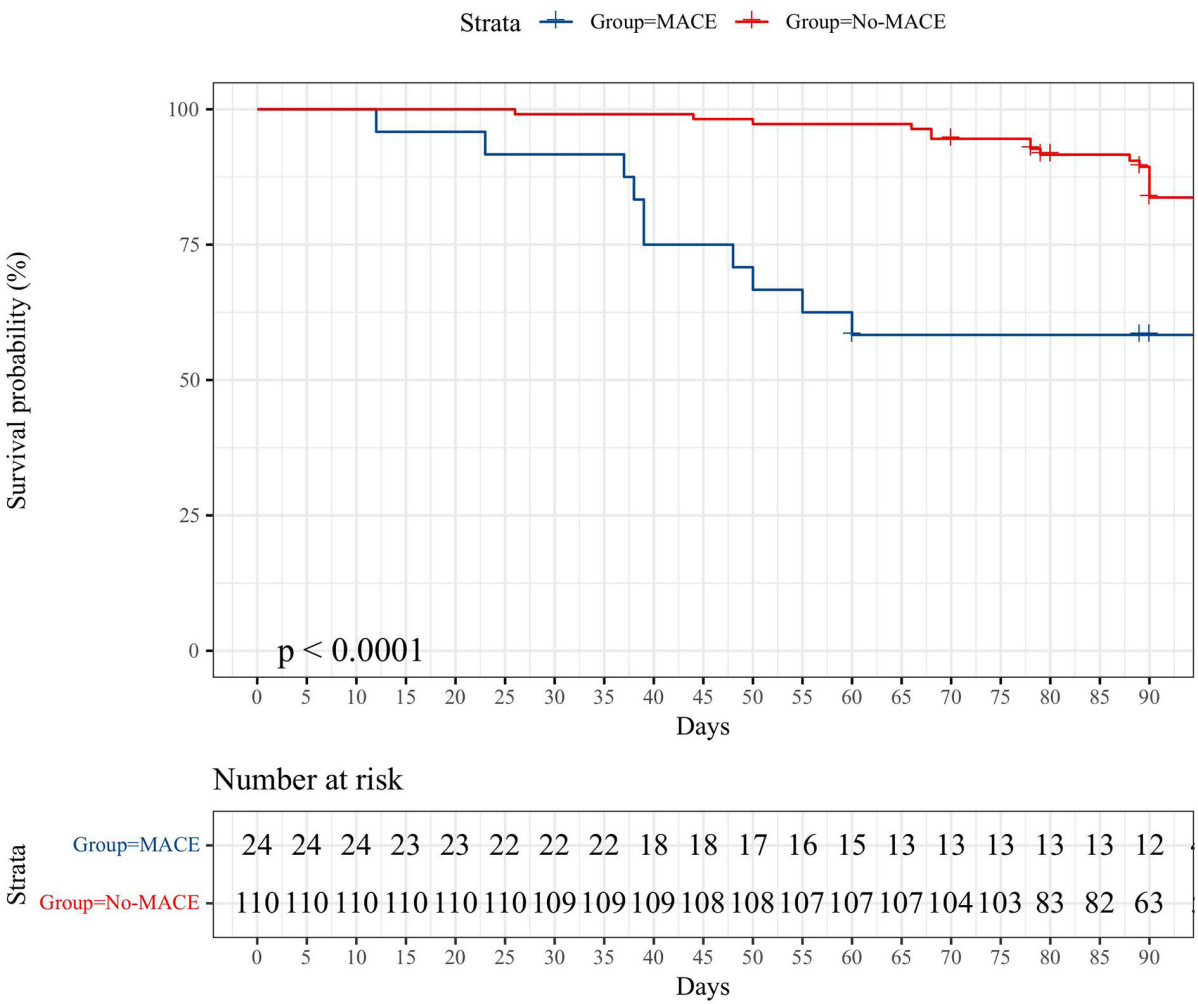
The K–M survival curve compares the 90-day all-cause deaths between the two groups.

The median follow-up of all cases was 90 days (12–102 days). In the MACE group, 13/24 of patients survived after careful treatment. The number of all-cause deaths in the MACE group was 12 (50%) as of the end of follow-up, and one of them was non-cardiogenic death (lung infection). For the non-MACE group, 16/110 (14.5%) of patients had non-cardiogenic deaths, and the rest were still alive at the end of follow-up. The K–M survival curves of the two groups are shown in Figure 2.

### Cardiac parameters among survivors and deceased patients in the MACE group

In the MACE group, 13/24 of patients survived after treatment, and 11/24 died after treatment. Compared with those of the survivors, the CK-MB (146.4 ± 56.2 vs. 77.3 ± 35.3, *p* ≤ 0.001), hsTNT [300.0 (218.5–729.0) vs. 112.0 (84.0–122.0), *p* ≤ 0.001], and NT-proBNP [8,400.0 (3,850.0–14,000.0) vs. 1,890.0 (1,200.0–2,400.0), *p* ≤ 0.001] levels of the deceased patients were significantly higher. Regarding the parameters of echocardiography, deceased patients had higher LVEDd (54.7 ± 4.7 vs. 48.7 ± 5.8, *p* ≤ 0.009) and lower LVEF (39.7 ± 6.4 vs. 52.6 ± 6.5, *p* ≤ 0.001) than those survivors. The QRS interval of the deceased patient group was significantly longer than that of the survivor groups (144.0 ± 37.5 vs. 113.1 ± 20.5, *p* = 0.020). There were no significant differences in other cardiac parameters between the two groups (Table 3).

**Table 3:** Cardiac parameters among survivors and deceased patients.

	**Survived (*N* = 13)**	**Deceased (*N* = 11)**	***P*-value**
Age, years	63.8 (7.7)	69.6 (7.6)	0.124
Male, *n* (%)	11 (84.6%)	7 (63.6%)	0.357
**Cardiovascular manifestations**			
Dyspnea	8 (61.5%)	9 (81.8%)	0.386
Edema	2 (15.4%)	2 (18.2%)	0.855
Palpitation	3 (23.1%)	0 (0.0%)	0.233
Chest pain	4 (30.8%)	1 (9.1%)	0.327
Days from first dose to onset	36.7 (20.4)	37.5 (21.0)	0.772
**Cardiac parameters**			
SBP, mmHg	124.5 (17.3)	113.1 (16.5)	0.111
DBP, mmHg	74.6 (10.2)	65.5 (15.6)	0.147
CK-MB, u/L	77.3 (35.3)	146.4 (56.2)	**0.003**
Cardiac troponin T, pg/mL	112.0 (84.0–122.0)	300.0 (218.5–729.0)	**0.001**
NT-proBNP, pg/mL	1,890.0 (1,200.0–2,400.0)	8,400.0 (3,850.0–14,000.0)	**0.002**
**Echocardiographic findings**			
LVEDd, mm	48.7 (5.8)	54.7 (4.7)	**0.009**
RVEDd, mm	32.1 (3.7)	34.2 (5.0)	0.222
LAESd, mm	35.9 (6.1)	39.1 (2.5)	0.130
RAESd, mm	32.9 (5.9)	34.1 (4.2)	0.662
LVEF, (%)	52.6 (6.5)	39.7 (6.4)	**< 0.001**
**ECG findings**			
Atrial fibrillation, *n* (%)	0 (0.0%)	1 (9.1%)	0.458
Advanced AV block, *n* (%)	3 (23.1%)	5 (45.5%)	0.390
Bundle branch block	2 (15.4%)	5 (45.5%)	0.182
FVP or VT, *n* (%)	4 (30.8%)	7 (63.6%)	0.107
PR interval, ms	173.9 (28.2)	161.7 (23.0)	0.234
Corrected QT interval, ms	471.2 (40.9)	469.7 (29.7)	0.977
QRS duration, ms	113.1 (20.5)	144.0 (37.5)	**0.020**

Data are (N) Mean (SD) or (N) n (%), Median (Q3–Q1), where N is the total number of patients with available data. For other abbreviations, see Table 2.

### The association between cardiac parameters and different outcomes

**Figure 3:**
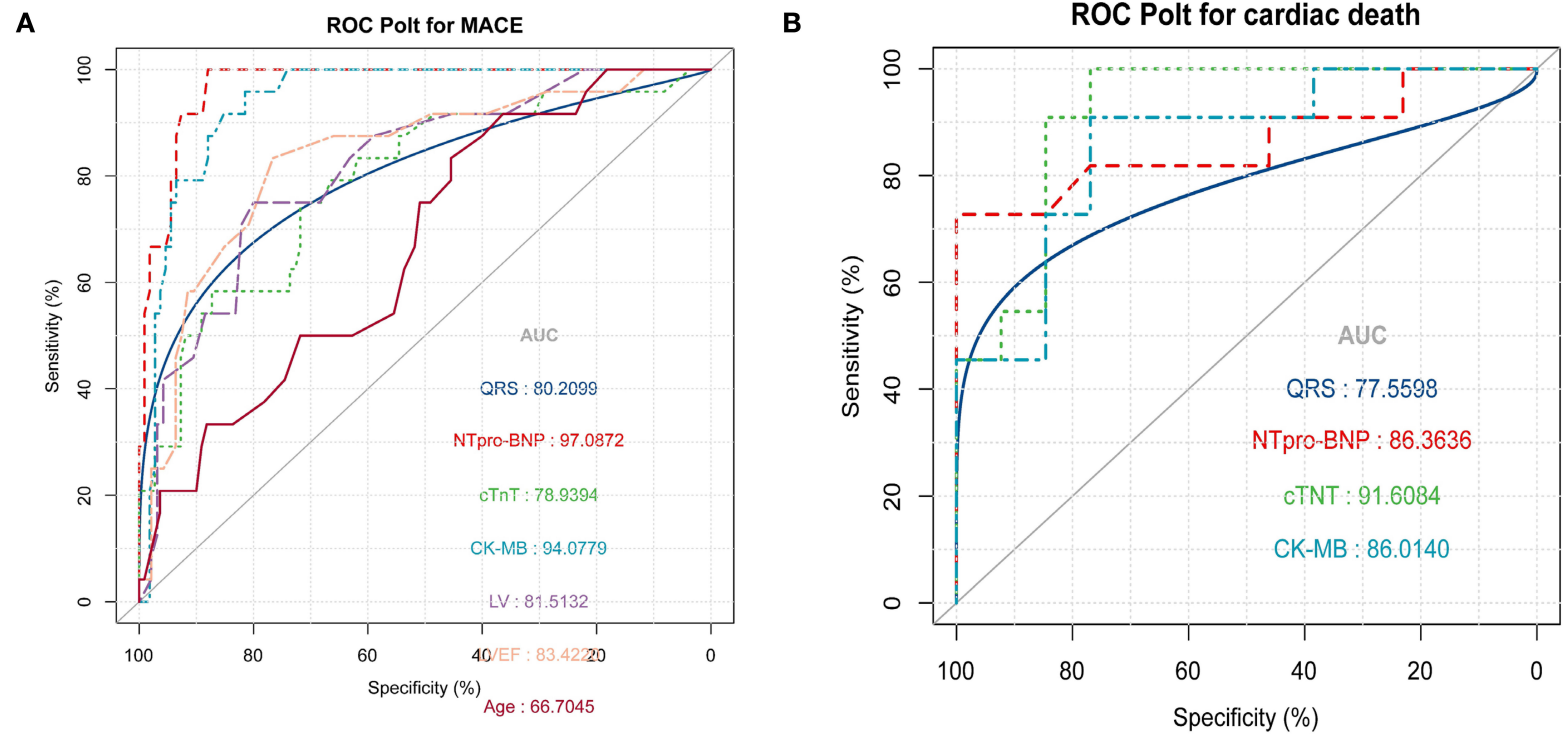
**(A)** Receiver operating characteristic (ROC) was used to compare the accuracy of various indicators to predict the occurrence of major adverse cardiac events (MACE) **(B)** ROC curve shows that high-sensitivity troponin T (hsTNT) is the most accurate marker for predicting cardiogenic death in the MACE group.

Univariate logistic regression was used to analyze the association between cardiac parameters and different outcomes. Age (*OR* = 1.08, 95% *CI* = 1.02–1.14, *p* = 0.007), CK-MB (*OR* = 1.03, 95% *CI* = 1.01–1.04, *p* < 0.001), hsTNT (*OR* = 1.01, 95% *CI* = 1–1.01, *p* = 0.001), NT-proBNP (*OR* = 1.0, 95% *CI* = 1.0–1, *p* < 0.001), LVEDd (*OR* = 1.21, 95% *CI* = 1.12–1.32, *p* < 0.001), LVEF (*OR* = 0.87, 95% *CI* = 0.82–0.93, *p* < 0.001), and QRS interval (*OR* = 1.04, 95% *CI* = 1.02–1.06, *p* < 0.001) were predictive of the development of cardiovascular symptoms (MACE events) in patients with PD-1 inhibitor-induced myocardial injury. The ROC curve was used to compare the accuracy of various indicators to predict the occurrence of MACE events. NT-ProBNP (*AUC* = 97.1) was the best predictor, followed by CK-MB (*AUC* = 94.1), LVEF (*AUC* = 83.4), LVEDd (*AUC* = 81.5), and other indicators, as shown in Figure 3A. In the MACE group, CK-MB (*OR* = 1.04, 95% *CI* = 1.01–1.07, *p* < 0.021), NT-proBNP (*OR* = 1.0, 95% *CI* = 1–1, *p* < 0.042), hsTNT (*OR* = 1.02, 95% *CI* = 1–1.04, *p* < 0.034), and QRS duration (*OR* = 1.04, 95% *CI* = 1–1.07, *p* < 0.032) were predictors of death. The ROC curve revealed that hsTNT was the most accurate predictive marker (*AUC* = 91.6; more details in Table 4 and Figure 3B).

**Table 4:** Logistic regression analysis of the association between cardiac parameters and different outcomes.

**Variables**	**MACE among 124 patients**	***p*-value**
	**OR (95% CI)**	
Age	1.08 (1.02~1.14)	**0.007**
Male	1.19 (0.43~3.34)	0.735
CK-MB	1.03 (1.01~1.04)	**< 0.001**
Cardiac troponin T	1.01 (1~1.01)	**0.001**
NT-proBNP	1.0 (1.00~1)	**< 0.001**
LVEDd, mm	1.21 (1.12~1.32)	**< 0.001**
RVEDd, mm	0.97 (0.87~1.07)	0.515
LAESd, mm	1.03 (0.95~1.11)	0.505
RAESd, mm	0.98 (0.89~1.06)	0.569
LVEF	0.87 (0.82~0.93)	**< 0.001**
PR interval	1 (0.98~1.01)	0.75
Corrected QT interval	1.01 (1~1.02)	0.093
QRS duration	1.04 (1.02~1.06)	**< 0.001**
**Cardiac death among 24 patients**		
Age	1.11(0.99~1.25)	0.086
Male	3.14 (0.45~21.96)	0.248
CK-MB	1.04 (1.01~1.07)	**0.021**
Cardiac troponin T	1.02 (1~1.04)	**0.034**
NT-proBNP	1 (1~1)	**0.042**
LVEDd, mm	0.87(0.7~1.07)	0.184
RVEDd, mm	1.13 (0.92~1.39)	0.24
LAESd, mm	1.17 (0.95~1.43)	0.135
RAESd, mm	1.05 (0.89~1.23)	0.57
LVEF	0.88 (0.75~1.04)	0.136
PR interval	0.98 (0.95~1.01)	0.258
Corrected QT interval	1 (0.98~1.02)	0.916
QRS duration	1.04 (1~1.07)	**0.032**

## Discussion

This is a retrospective case analysis from a large referral hospital. We are deeply concerned about the increase in high-sensitivity troponin T levels after PD-1 inhibitor treatment, and previous studies have reported that the incidence is very low. In the safety study of more than 2,000 patients with immunotherapy released by Bristol–Myers Squibb, the rate of myocarditis in patients treated with ipilimumab or nivolumab was 0.09%. Among patients receiving combination therapy, the incidence of myocarditis is approximately 0.3%, and its severity is greater than that of patients receiving monotherapy (8). A retrospective case study of PD-1 inhibitor treatment reported that the prevalence of myocarditis was 1.14% with a median time of onset of 34 days after starting PD-1 inhibitor treatment (*IQR*: 21–75 days) (13). Since many patients do not routinely have ECG and markers of myocardial injury monitored, some studies suggest or believe that the proportion of myocarditis caused by PD-1 inhibitors may be higher than 1% (7). However, in clinical practice, we often encounter patients who show only elevated levels of cardiac troponin, a marker of myocardial injury but have no symptoms after treatment with PD-1 inhibitors. These patients have not been well evaluated. Our study showed that 3.48% (134/3848) of patients had increased high-sensitivity troponin T levels after PD-1 inhibitor monotherapy. This ratio is very high and still underestimated because some patients without cardiovascular symptoms have not been monitored for troponin levels. Our study indicated that 24 patients (0.62%) had cardiovascular symptoms, and 11 of them suffered cardiogenic death. If these symptomatic patients are defined as having myocarditis, this is equivalent to the incidence rate of previous studies.

Our study supports the need for routine monitoring of cardiac parameters in patients using PD-1 inhibitors. Oncologists in many countries currently recommend routine detection of myocardial injury markers, such as CK-MB, CK, troponin, and BNT-proBNP, during each cycle of PD-1 inhibitors (14, 15). However, the importance of the elevated levels of each marker is unclear, and cardiovascular physicians often go to the oncology department for consultation. Our study indicates that the higher the increase in these cardiac markers levels, the greater the probability of occurrence of cardiac symptoms. In addition, our study indicates that the QRS interval on ECG is also a clinical indicator for predicting whether patients will have symptoms, which suggests that ECG is also very important in monitoring patients for adverse drug reactions. This is similar to a previous study by Zlotoff et al., which showed that the QRS duration is increased in ICI myocarditis and is associated with increased MACE risk, especially in patients whose QRS interval is greater than 110 ms (16). We think this is mainly related to the occurrence of more ventricular arrhythmias in the MACE group. With an increasing number of ventricular arrhythmias, the probability of cardiovascular symptoms will obviously increase. Of course, cardiac ultrasound is a very accurate tool to judge whether a patient has cardiac dysfunction, especially the LVEF is a very important indicator. However, using the ROC curve for comparison, NT-proBNP is the best cardiac parameter predicting clinical symptoms in patients with PD-1 inhibitor-mediated myocardial injury. This may be related to the fact that most patients in the MACE group present with symptoms of HF.

Our study indicated that the most common occurrence of cardiovascular system symptoms after PD-1 inhibitor treatment is HF symptoms, manifested as dyspnea and edema. Then, five patients presented with chest pain, four patients experienced palpitations, and one patient died suddenly after elevated troponin levels were observed. Notably, 62.5% (16/24) of patients in the MACE group entered the intensive care unit for treatment. However, 45.8% (11/24) of the patients eventually experienced cardiogenic death. Such a high mortality rate is similar to that reported in Western countries (17–19). Additionally, the proportion of corticosteroid treatment was relatively low compared to that in Western countries; 23 patients in the MACE group received treatment, and 13 patients received glucocorticoids. A recent study (20) showed that the dose of corticosteroids is negatively correlated with the mortality of patients with PD-1 inhibitor-mediated myocarditis. However, these results increase the possibility that myocardial injury can be mitigated by early and intensive corticosteroid therapy. Nevertheless, the decision of whether to administer high-dose corticosteroids during clinical practice still requires consideration of various other aspects, especially infection. Certainly, we cannot rule out that this mortality rate is related to the conservative use of corticosteroid therapy. Despite the high mortality rate, we still need to risk stratify patients. We also used a logistic model to evaluate the relationship between various cardiac parameters and cardiogenic death. CK-MB, hsTNT, NT-proBNP, and QRS duration were statistically significant in predicting cardiogenic death in the MACE group. Using ROC curves for mutual comparison, hsTNT was the best marker for predicting cardiogenic death in the MACE group patients.

In 2018, the American Society for Clinical Oncology (ASCO) issued the clinical practice guidelines (21, 22) for cardiotoxicity related to ICIs. Based on this guideline, cardiotoxicity is divided into four levels according to severity (23). Patients who exhibit only increased levels of markers of myocardial injury without any symptoms are divided into 1 level and do not need corticosteroid treatment, however, monitoring of cardiac parameters needs to be continued. The results of this study may help clinicians identify, early in the course of the disease, which patients with level 1 will continue to develop symptoms and which patients with symptoms will continue to progress to death. In view of the very high mortality rate of PD-1 inhibitor-related myocarditis, these results may help us to stay aware of specific patients and provide more appropriate treatments in the early stages of disease deterioration.

This study also has some limitations. First of all, this is a single-center retrospective study. Although we want to clarify the specific probability of myocardial injury after PD-1 inhibitor treatment, a large proportion of the data is incomplete, and there are many deviations. Laboratory indicators and ECG indicators are complete, but there are missing data on cardiac ultrasound. Thus, we used the mean instead. This led to a shift in the research results. Second, we cannot completely rule out myocardial damage caused by other drugs, such as chemotherapy drugs, such as paclitaxel and platinum, although these drugs are rarely reported to cause myocardial damage, at the same time, we cannot completely rule out myocardial infarction, stress cardiomyopathy, and other causes of myocardial injury in these patients because of the lack of very complete clinical examination results. Third, the small sample size and information bias may affect the results of our study. Further research should be conducted with larger sample size and minimize the information bias for more reliable results.

## Conclusion

In patients with myocardial injury after PD-1 inhibitor treatment, NT-proBNP is the superior parameter of choice to predict the likelihood of developing cardiovascular symptoms, whereas, in symptomatic patients, hsTnT is superior to other cardiac parameters and is associated with the development of death.

## Data availability statement

The raw data supporting the conclusions of this article will be made available by the authors, without undue reservation.

## Ethics statement

The studies involving human participants were reviewed and approved by Human Research Committee of the Second Xiangya Hospital of Central South University. The patients/participants provided their written informed consent to participate in this study.

## Author contributions

JL designed this study and performed quality control of data authenticity. XP drafted the manuscript and collected and analyzed the data. ZZ, NL, SZ, YZ, and JL revised the paper. All authors approved the final version.

## Funding

This research was supported by the National Natural Science Foundation of China (82100314).

## Conflict of interest

The authors declare that the research was conducted in the absence of any commercial or financial relationships that could be construed as a potential conflict of interest.

## Publisher's note

All claims expressed in this article are solely those of the authors and do not necessarily represent those of their affiliated organizations, or those of the publisher, the editors and the reviewers. Any product that may be evaluated in this article, or claim that may be made by its manufacturer, is not guaranteed or endorsed by the publisher.
